# Sources of anxiety in young rural physicians working alone on remote islands: A qualitative study

**DOI:** 10.1002/jgf2.488

**Published:** 2021-08-02

**Authors:** Kaku Kuroda, Moe Kuroda, Ryuichi Ohta

**Affiliations:** ^1^ Department of Family Medicine SUNY Upstate Medical University Syracuse NY USA; ^2^ Department of General Medicine University of Toyama Toyama Toyama prefecture Japan; ^3^ Community Care Unnan City Hospital Unnan Shimane prefecture Japan

**Keywords:** anxiety, remote islands, rural medicine, solo practice, young physicians

## Abstract

**Background:**

We aimed to examine the sources of anxiety for young rural physicians working alone on remote islands.

**Methods:**

Semistructured interviews were conducted on six physicians who worked on remote islands. The Steps for Coding and Theorization method was used to analyze the content of the interviews.

**Results:**

Twelve concepts were generated and categorized into four themes: solo practice, the tight‐knit community, limited human and medical resources, and future career.

**Conclusion:**

Young rural physicians' anxieties in solo practice on a remote island are complicated and include multiple dilemmas. Recognizing these anxieties helps with metacognition and professional development in these individuals.

## 1 BACKGROUND

Solo rural practice has many well‐known disadvantages, including overwork and the physician's dilemma because of isolation.^1^ However, rural areas are still heavily dependent on solo practitioners.^2^

The family medicine residency program in Okinawa, Japan, includes a unique clinical training that requires young physicians in their fourth or fifth postgraduate year (PGY) to work alone on remote islands.^3^ The goal of this program is to simultaneously provide essential experience to young physicians, as well as to ensure that these islands, that face a lack of doctors, have a physician. Okinawa has sixteen prefectural clinics on each island where there is only one allocated physician and one nurse. Hence, it is their responsibility to address all the medical needs of the inhabitants, including all after‐hour calls, which can become a source of anxiety.

Understanding the anxiety suffered by physicians practicing in rural areas may help ease their mental burden. Moreover, analyzing the anxiety of young rural physicians in solo practices may allow for professional development, improving the quality of rural medicine.

This study aimed to examine the anxiety of young rural physicians working alone on remote islands.

## 2 METHODS

A qualitative research was performed with six physicians who were residents in the family medicine program in A Hospital and had completed their 3‐year postgraduate clinical training with general rotation in all specialties. All participants were recruited from clinics in one of the sixteen islands. We excluded those who had prior solo‐practice experience, in order to focus on the anxiety faced by new, young solo practitioners. Sampling was concluded upon completion of interviews of the voluntary research participants.

We conducted one‐on‐one, semistructured interviews online between January 2018 and February 2018. The interviews followed the interview guide as shown in Table 1. Each participant was interviewed only once.

**TABLE 1:** Interview guide

No	Question
1	What were your anxieties before going to work on a remote island?
2	What were the new anxieties that you started to feel after going to a remote island?
3	Is there anything else that you thought you should have been more prepared for before going to a remote island?
4	Are there any other measures that we could take to relieve these anxieties?

The interviews were tape‐recorded and transcribed verbatim. The data were analyzed using the Steps for Coding and Theorization (SCAT) method for analysis.^4^, ^5^ In the process of SCAT, concepts were extracted from interview data and were categorized into themes. The main author conducted those processes initially before the second and third authors reviewed the coding. This was followed by triangulation. When opinions regarding the coding differed, the three authors discussed the issues and agreed on the final concepts and themes. The research paradigm is based on the theory of constructivism.

We obtained written informed consent from all participants prior to the study. The Ethics Committee of B Hospital approved this study.

## 3 RESULTS

The average age of the six participants was 30.2 ± 2.67 years. All participants were males and were either in their fourth or fifth PGY. The average duration of the interview was 48.2 ± 3.76 min.

Twelve concepts were generated and categorized into four themes as shown in Table 2.

**TABLE 2:** Results of the qualitative analysis

Categories	Concepts	Quotations
Anxiety related to solo practice	Anxiety over an extremely wide scope of practice	*I am most worried about how to handle CPA* a *and respiratory failure alone. I'm anxious about whether I can take on the leadership role to mobilize my staff*.
	Pressures of working unreasonably long hours	*There is no way to escape, both in good and bad ways. Sometimes I feel like I am under house arrest. Sometimes when I am really busy, it feels like an emergency room. Sometimes when this happens, I really want to rest, but I do not always have the luxury to take the day off*.
	Sense of responsibility over subjective medical care not monitored by external observers	*I wonder if my diagnoses are correct. I can only diagnose based on a medical interview and physical examination, however, sometimes I question the validity of my diagnoses. There is no way to know if it was really caused by alcohol, so I am unsure if my diagnostic skills are really improving; there is no way to assess that*.
Anxiety related to the tight‐knit community	Conflicts associated with simultaneous physician‐patient and neighborly relationships with residents	*It is the only clinic on the island, therefore I am unsure to what extent I should behave as the physician or just as a member of the island community at village events and interpersonal communications with town residents. Where do I draw the line between my professional and personal life in my daily encounters? Other doctors seem to be managing this skillfully, but I am unsure because there's no framework for guidance*.
	Close interpersonal relationships within a small, insular community	*Now that I have developed relationships with my patients over my 2‐year post, I experience more anxiety from the pressure. It is great when my practice results have good outcomes, but there is an enormous amount of mental stress when they lead to bad ones. It is not like when I worked at the hospital and my patients were short‐term encounters. Here, I have personal relationships with them. It makes me want to do good for my patients, which doubles my anxiety when the management I prescribe leads to bad outcomes*.
	Sense of duty arising from affinity with the community	*They often tell me that I can drink today because I would not be called. However, I wonder, what if I do get called while completely wasted, that would be a problem. Moreover, if I keep declining their invitations, they will think I am cold. I get invited to all sorts of things, like fishing on the weekend, but I question how appropriate it would be to develop closer relationships with specific individuals. Then, would I be able to practice care appropriately if something happened to any of them? I am always extra cautious in managing the subtle interpersonal dynamics and [appropriate] distance*.
Anxiety related to limited human and medical resources	Leadership demanded from the only physician in the multidisciplinary team	*There are many things that were previously handled by my multidisciplinary care partners in large hospital settings. For example, social workers made the discharge arrangements and care managers engaged in the subtle discussions with patients' families. Of course, these things are stipulated under certain frameworks, but there are instances when the clinic physician became too forward (for example, when deciding who take the initiative in a given conference). I am never certain about the level of openness that meeting attendees experience or how fair the moderator is running the discussions. It is often taken for granted that the physician leads the discussions, but I wonder if that is correct, if it is normal that the discussion does not go anywhere without my intervention. I am also uncertain about how cooperation plays out and how to take the initiative because I am not used to it*.
	Comprehensive care with knowledge of the medical and welfare systems on the remote island	*It has been quite a while since I studied about policies for the national medical licensing exam, so I have forgotten, for example, how long‐term care insurance works. While I was a resident, it only came up when a patient in the general ward was transferred (or some similar situation); I was more focused on medical aspects*.
	Judgment on level of urgency and when and whether to use emergency transport with understanding of environmental conditions on the remote island	*I realized that it was hard to make the decision whether to transport a patient to hospital. I would not hesitate if it was a clear case that required helicopter transfer, but in a case, for example, the condition of a heart failure patient arriving in the evening worsens suddenly, do I inject Lasix and wait for one night at the clinic or call for a helicopter immediately because it is too dangerous? Another example would be an elderly patient with flail chest from trauma, but no hemothorax and pneumothorax. I hesitate in situations like this and wonder what to do overnight, but I also guess that even if the patient were to be transferred via helicopter, he/she would not be operated on anyway*.
	Negative emotions of tourists who have little understanding of the medical resources on the remote island	*Cases involving tourists are surprisingly nerve‐racking. There was a tourist who almost drowned in September. He was intubated and transported by helicopter, but I was very scared, wondering what would have happened if I had not been able to intubate him properly and let him die in front of my eyes. I cannot help feeling more nervous about tourists than residents, especially when their conditions are serious*.
Anxiety related to the future career	Vanity from being given social status incongruent with the level of experience or skill	*It is strange to be a third‐year doctor and also the clinic director. While on the island, everyone treats me with respect. As the only doctor, I am treated like a special guest and I am invited to attend events with other people in positions of authority on the island, such as school principals. Now, I am weirdly used to it, but it still makes me feel anxious. I do not know if this position matches my level of experience or lack thereof. It feels so awkward that I am the only doctor here, in the fourth year after graduation, with the title of clinic director. I wonder if it is really okay for me to fill this position because of my lack of experience; that is a source of my anxiety as well*.
	Limitation of career options after completing the post on a remote island	*I am scared that my skills will deteriorate after two years of being here. I sometimes wonder what I will do once I complete this post on the remote island and whether I should undergo additional training*.
Story line		
It is inevitable that all young physicians posted on remote islands will experience “anxiety related to solo practice,” which has its roots in [anxiety over an extremely wide scope of practice], including emergency care. They are also placed under great [pressures of working unreasonably long hours] because they have no replacement staff. The [sense of responsibility over subjective medical care not monitored by external observers] also arises from practicing alone as young physicians with limited experience. They experience great “anxiety related to the tight‐knit community” in their [close interpersonal relationships within a small, insular community]. While they experience [conflicts associated with simultaneous physician‐patient and neighborly relationships with residents] as the sole physicians on the islands on which they reside, their [sense of duty arising from affinity with the community] is fostered within the narrow community. They also face “anxiety related to limited human and medical resources” in their isolated situations. Young physicians must confront the expectations of [leadership demanded from the only physician in the multidisciplinary team] and strive for [comprehensive care with knowledge of the medical and welfare systems on a remote island] within the limited environment. In emergencies, they are required to make [judgments on the level of urgency and when and whether to use emergency transport from a remote island]; in situations involving many tourists, they hold [negative emotions toward tourists who have little understanding of the medical resources on a remote island]. They also struggle with “anxiety related to the future” because of being young but also as the sole physician on an island, the state of which was characterized by [vanity from being given social status incongruent with one's level of experience or skill] and the [limitation of career options after the post on a remote island].		

^a^ Cardiopulmonary arrest.

### 3.1 Solo practice

Participants reported feeling anxious about handling all fields of medicine alone. Diagnostic competency and decision making about to what extent they should care were important, considering the poor access to specialists.

All physicians also expressed anxiety about caring for all cases, mild to severe, during 24‐h shifts, even on weekends and holidays, with a conflict between their sense of duty and perseverance as the sole physician.

They raised concerns about not receiving any guidance from third parties because, during their island posts, they typically did not have opportunities to work with other physicians.

### 3.2 The tight‐knit community

Physicians experienced anxiety about whether their professionalism was being compromised when the physician‐patient relationship was obscured by their status as a fellow island resident, as well as conflicts surrounding their sense of mission.

How the island residents viewed them was a source of anxiety because the sole physician must maintain close interpersonal, long‐term relationships with everyone within an insular environment.

### 3.3 Limited human and medical resources

The physicians raised concerns about the need to fill leadership roles as the only physician on the island, to remain in harmony with the stakeholders in closeness with other professionals, and to maintain a good understanding of policies and services that can be applied adequately on remote islands in order to provide comprehensive care.

Emergency cases require requesting helicopters. Deciding when to transport patients is also at the physician's discretion. Some cases that arise during the night are kept at the clinic for observation until the following morning. Physicians mentioned anxiety about performing appropriate triage and making adequate judgment calls with limited access to medical services.

The concern about after‐hours care of tourists is valid because tourists are not always aware of these limitations and often expect care that goes beyond what is provided with the limited resources available.

### 3.4 Future career

The participants felt anxious about the incongruence between their status and treatment they received as sole physicians versus their actual level of experience or competency.

Physicians were also anxious that they might have lowered their skills in handling cases that did not arise in outpatient conditions on the islands.

## 4 DISCUSSION

We generated important concepts about young physicians' anxiety on remote islands for improving the professional development process.

The generated concepts demonstrated a complicated structure involving multiple dilemmas. Negative emotions were gradually controlled after reflecting with other medical professionals and patients over time, leading to realizations of their own growth.^6^ Therefore, more frequent online reflections with other physicians and more experience with patients should be required for young solo practitioners.

Anxiety about limited resources appeared when the physicians initially started working, but this disappeared gradually with increased interprofessional collaboration and a family‐oriented approach in blending into the rural community. These qualities were described as essential principles of family medicine.^7^ This suggests that the experiences they underwent were good opportunities to learn family medicine because facing these anxieties could help family physicians overcome difficult situations in their daily practice.

Physicians' self‐awareness in practice helps them develop essential qualities to understand patients.^8^ Therefore, recognition and reflection of anxiety in young rural physicians were significantly meaningful for metacognition and building resilience.^9^, ^10^

Two limitations should be acknowledged. First, as the interviewer was also the only physician on a remote island, some anxieties in rural practice might have been omitted in the interview as tacitly understood. However, some of the new knowledge was obtained because the interviewer understood their backgrounds. Hence, the results are not entirely limited by reflexivity and have satisfactory validity. Second, sample size is relatively small in this study. The following study can investigate more participants from varied backgrounds.

## 5 CONCLUSION

Our study found that young rural physicians' anxieties in solo practice on remote islands were complicated and included many dilemmas. Recognizing these anxieties helps with metacognition and professional development in young physicians.

## CONFLICT OF INTEREST

The authors have stated explicitly that there are no conflicts of interest in connection with this article.
